# Establishing a risk stratification model to identify clinically high-risk N0 breast cancer who could benefit from regional nodal irradiation: a single institute analysis

**DOI:** 10.3389/fonc.2024.1290852

**Published:** 2024-09-13

**Authors:** Wei-Xiang Qi, Lu Cao, Dan Ou, Shuyan Li, Cheng Xu, Rong Cai, Haoping Xu, Gang Cai, Jiayi Chen

**Affiliations:** ^1^ Department of Radiation Oncology, Ruijin Hospital, Shanghai Jiaotong University School of Medicine, Shanghai, China; ^2^ Shanghai Key Laboratory of Proton-therapy, Ruijin Hospital, Shanghai Jiaotong University School of Medicine, Shanghai, China

**Keywords:** breast cancer, risk stratification, beast conserving surgery, whole breast irradiation, regional nodal irradiation

## Abstract

**Background:**

The purpose of this real-world study was to investigate the risk factors for developing recurrence among patients with pathological T1-3N0 breast cancer (BC) treated with breast-conserving surgery (BCS) followed by whole breast irradiation alone (WBI) and identify those clinically high-risk BCs who could benefit from regional nodal irradiation (RNI).

**Materials and methods:**

Female BC patients treated at Shanghai Ruijin hospital from 2009 to 2016 were retrospectively reviewed. The disease-free survival (DFS), breast cancer specific survival (BCSS) and overall survival (OS) were estimated by the Kaplan-Meier method, and survival differences were compared with the log-rank test. Univariate and multivariate analysis was performed using Cox proportional hazards regression analysis. An external validation was conducted by using SEER database.

**Results:**

A total of 622 BC patients treated with BCS+WBI alone were included. With a median follow-up of 82 months, the 7-year OS, BCSS and DFS for the entire cohort was 97%, 99% and 91%, respectively. Multivariable Cox analysis indicated that tumor size (*p*=0.006), tumor location (*p*=0.033), lymphovascular invasion (LVI) status (*p*=0.0028) and Ki-67 index (*p*=0.051) were independent risk factors for DFS. A scoring system was developed using these four factors and the 7-year DFS and OS were 97% and 96% for patients with 0-1 risk factors, 95% and 82% for patients with ≥2 risk factors (*p*<0.0001 for DFS, and *p*=0.0063 for OS). Based on tumor size and tumor location, an external validation by demonstrated that the 7-year OS was 90% and 88% for patients with 0-1 risk factor, which was significantly better than those defined as high-risk BC patients (82%, *p*<0.0001).

**Conclusion:**

By using our institute database, we establish a risk stratification system for identifying sub-group of pN0 BC patients, who are at high risk for developing recurrence. The results of our study support tailored RT decision-making according to individual risks, which needed to be confirmed in further studies.

## Introduction

According to the mostly recent publication from National Cancer Center (NCC) of China, breast cancer(BC) is the most commonly diagnosed cancer in women, accounting for 16.72% (306,000) of all new cancer cases in 2022 (1). In a multi-center, hospital-based observational study, early-stage BC (stage I and II) accounts for 78.3% of all BC cases in China (2). It has been established that radiation therapy plays an important role in the multidisciplinary management of early-stage BC after breast conserving surgery (BCS) (3–5). Randomized studies provide evidence that BCS combined with WBI results in long-term overall survival comparable to modified radical mastectomy (6, 7). Results from individual meta-analysis performed by Early Breast Cancer Trialists’ Collaborative Group (EBCTCG) also indicates that radiation therapy (RT) after breast-conserving surgery (BCS) could reduce local recurrence (5-year local recurrence reduction 19%) and breast cancer mortality (*p*=0.005) (8). Based on these high-quality data, whole breast irradiation (WBI) ± boost to tumor bed followed by BCS is the established treatment for pN0 BC patients according to the National Comprehensive Cancer Network (NCCN) guideline (9). However, pN0 breast cancer is a heterogeneous disease that encompasses different clinical behaviors and responses to therapy, the optimal radiation volume for early-stage BC patients remains controversial. MA.20 study is a large phase III trial to investigate the potentially survival benefit from the addition of regional nodal irradiation (RNI) to WBI among early-stage BC patients after BCS. In this study, a total of 177 “high-risk” node-negative breast cancer defined as a primary tumor measuring 5 cm or more or 2 cm or more with fewer than 10 axillary nodes removed and at least one of the following: grade 3 histologic categorization, estrogen-receptor (ER) negativity, or lymphovascular invasion (LVI) are also included for analysis, and sub-group analysis indicates that RNI+WBI has a tendency to improve 10-year DFS when compare to WBI alone (72.4% vs. 83.7%, HR 0.55, 95%CI: 0.28-1.09) (10). In another large phase III trial assessing efficacy of RNI in early-stage BC patients, a sub-group of 1778 clinically “high-risk” pN0 BC, defining as centrally or medially located primary tumor, are included for analysis. The result shows that RNI also improves overall survival (HR0.79, 95%CI: 0.61-1.02) when compared to controls (11). In a more recent report, the long-term outcomes from EORTC-22922 trial confirms that RNI has a tendency to improve the OS among pN0 BC patients when compared to controls (HR 0.88, 95%CI: 0.72-1.08) (12). Based on these result, NCCN guide recommend RNI in those clinically “high risk” pN0 patients, defining as central/medial tumors or tumors >2 cm with other high-risk features (young age or LVI). However, to our best knowledge, the adverse clinical features for pN0 BC patients significantly varies and there is no established clinical risk stratification system to identify those “high-risk” pN0 BC patients who might benefit from RNI. As a result, we perform the present study to establish a risk stratification system for identifying sub-group of pN0 BC patients treated with BCS+WBI alone, who are still at high risk for developing recurrence. And stratify the subgroup who regional nodal irradiation might be indicated.

## Materials and methods

### Patients

**Figure 1:**
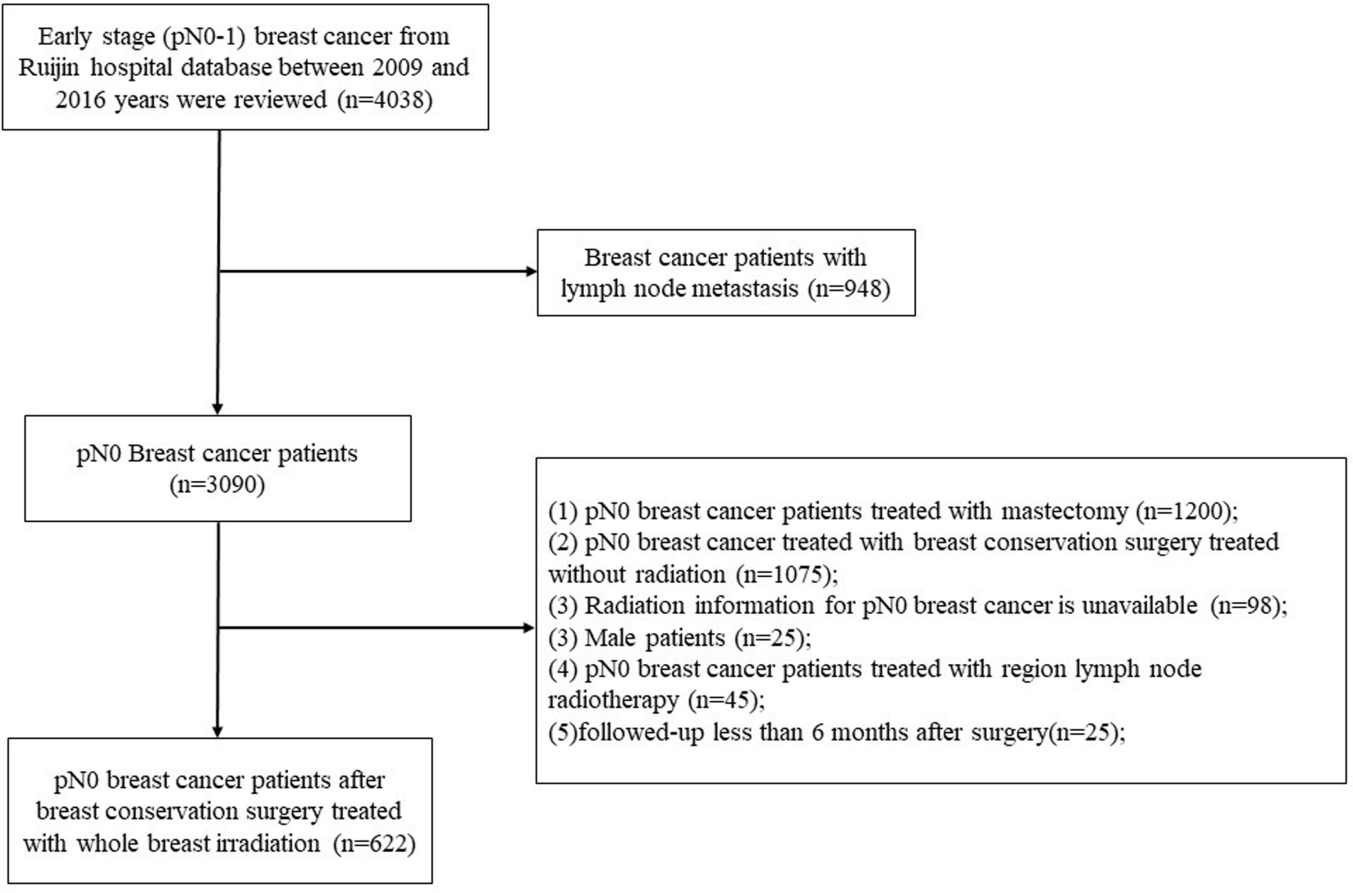
Flow diagram of included patients.

Clinical information of invasive breast cancer who underwent BCS and axillary dissection or sentinel node biopsy followed by WBI alone between January 2009 and December 2016 in Shanghai Ruijin Hospital were retrospectively reviewed. Eligible criteria were as follows: female, age ≥ 18 years, no distant metastasis, no neoadjuvant treatment, complete tumor resection (margins ≥1 mm), histologically confirmed primary BC with negative axillary nodes metastasis, and treated with BCS followed by WBI alone. The exclusion criteria were as follows: bilateral invasive breast cancer, pN0 breast cancer treated with BCS without WBI, detailed radiation information of treatment volume was unavailable, patients undergone postoperative WBI+RNI or follow-up less than 6 month after surgery. After selection, a total of 622 patients with pN0 BC were eligible for analyses (**Figure 1**).

The following data were collected: age at diagnosis, gender, date of surgery, tumor size, histology, tumor grade, lymphovascular invasion (LVI) and nodal status, hormone receptor status including estrogen receptor (ER) and progesterone receptor (PR); and human epidermal growth factor receptor 2 (HER2), and treatment regimens such as type of definitive surgery, chemotherapy, targeted therapy, endocrine therapy and volume of adjuvant radiotherapy. The present study procedures were approved by the Ethical Committee of RuiJin Hospital affiliated medicine school of Shanghai Jiao Tong University.

### External validation cohort

We used all 18 registries of the SEER database to identify patients diagnosed with breast cancer in the period from January 2000 to December 2018.We obtained permission to access the data files from the SEER program by NCI with the reference number 12363-Nov2021. Using primary tumor site of the breast, behavior code ICD-0-3 “malignant”, AJCC N stage=N0, AJCC M stage=M0, therapy radiation recode = “beam radiation” and therapy Surg/Rad Seq= “radiation after surgery”, we identified 59,173 BC patients. After excluding BC patients treated with mastectomy, or neoadjuvant systematic therapy or primary tumor location of breast is unknown or overlapping lesion of breast, a total of 24,029 BC patients treated with BCS and adjuvant radiation were included for analysis.

### Outcomes definitions

The primary endpoint of the current analysis was disease-free survival (DFS) defining as the time from surgery to the time of a first recurrence in the ipsilateral breast or in nodal or distant sites, a contralateral breast cancer, or death from breast cancer, which was in consistent with definition of DFS in MA-20 trial (10). The secondary endpoint was overall survival (OS) and breast cancer specific survival (BCSS). OS was defined as the time from surgery to death from any cause. BCSS was defined as the time from surgery to death from breast cancer.

### Statistical analysis

Descript analysis was used to summarize the baseline characteristics. Continuous variables were summarized by median and range, and qualitative variables were described as frequencies and proportion. Univariate and multivariate Cox proportional hazards regression analysis were performed to identify independent risk factors for DFS, and variables with a p-value < 0.1 in univariate analysis were included for Cox stepwise proportional hazards regression analysis. Survival curves for DFS and OS were estimated using the Kaplan-Meier method and a 2-sided log-rank test stratified by number of risk factors was performed to compare survival difference between low-risk and high-risk groups. All statistical analyses were performed by using NCSS 11 Statistical Software (2016) (NCSS, LLC. Kaysville, Utah, USA, ncss.com/software/ncss) and MedCalc Statistical Software version 15.2.2 (MedCalc Software bvba, Ostend, Belgium; http://www.medcalc.org; 2015).

## Results

### Baseline characteristics

The patient characteristics and treatment information were summarized in **Table 1**. The median age was 53 years (range, 25–83 years). Of all patients, most patients had tumors categorized as T1(79.4%), ER positive (75.9%), HER-2 negative (88.7%), LVI negative (89.5%), Grade I-II (60.6%) and histologic type of invasive ductal carcinoma (IDC,87.3%). Nearly half of included patients (54.3%) received adjuvant chemotherapy. Among the 338 patients who received adjuvant chemotherapy, anthracycline- and/or paclitaxel-based regimens were used in 280 patients (82.8%). There were 472 patients with ER positive BC, 95% of them treated with endocrine therapy. A total of 46 (68.6%) out of 67 patients with HER-2-positive disease received trastuzumab. As for the regimen of radiotherapy, the majority of included patients (95.3%) received WBI with tumor bed boost.

**Table 1:** baseline characteristics of included patients.

Characteristics	N, %
**Age, median**	53, (25-83) years
**≤50**	233 (37.5%)
**>50**	389 (62.5%)
Tumor size	
**≤2cm**	494 (79.4%)
** >2cm**	128 (20.6%)
Pathological T stage	
**pT1**	494 (79.4%)
**pT2**	123 (19.8)
**pT3**	5 (0.8%)
ER status,	
**positive**	472 (75.9%)
**negative**	150 (24.1%)
HER-2 status	
**positive**	67 (10.8%)
**negative**	552 (88.7%)
**unknown**	3 (0.5%)
Tumor location	
**External**	396 (63.7%)
**Centrally or medially**	266 (36.3%)
LVI status	
**positive**	55 (8.8%)
**negative**	557 (89.5%)
**unknown**	10 (1.6%)
Number of axillary nodes dissected	
**<10**	534 (85.9%)
**≥10**	88 (14.1%)
Ki-67 index	
**≤14%**	267 (42.9%)
**>14%**	353 (56.8%)
**Unknown**	2 (0.3%)
Grade	
**Grade I-II**	377 (60.6%)
**Grade III**	219 (35.2%)
**Unknown**	26 (4.2%)
Histology	
**IDC**	543 (87.3%)
**Others**	79 (12.7%)
Endocrine therapy	
**yes**	448 (72.0%)
**no**	127 (20.4%)
**unknown**	47 (7.6%)
Chemotherapy	
**yes**	338 (54.3%)
**no**	257 (41.3%)
**unknown**	27 (4.3%)
Regimen of adjuvant radiotherapy	
**WBI with tumor bed boost**	593 (95.3%)
**WBI without tumor bed boost**	29 (4.7%)
Anti-HER2 therapy among HER-2 positive BC	
**Yes**	63(94%)
**No**	5 (6%)

IDC, invasive ductal carcinoma; BC, breast cancer; IDC, invasive ductal carcinoma; LVI, lymphovascular invasion.

### Survival analysis

The detailed pattern of recurrences and deaths were provided in **Table 2**. A total of 14 patients developed isolated locoregional recurrence and 18 patients with distant recurrence. A total of 13 patients died, majority of them (61.5%) died from breast cancer. By the latest follow-up of Oct 2022, with a median followed-up of 82 months, a total of 20 (3.2%) patients died in the entire cohort, with 10 (1.6%) patients died from breast cancer. The 7-year DFS, OS and BCSS was 91%, 97% and 99%, respectively.

**Table 2:** disease recurrence or death.

Event	Number of patients (%)
Isolated locoregional recurrence	14 (2.3)
Local (in breast) only	7 (1.1)
Regional only	2 (0.3)
Local and regional	5 (0.8)
Distant recurrence	18 (2.9)
Distant alone	13 (2.1)
Concurrent with locoregional recurrence	5 (0.8)
Any recurrence or contralateral breast cancer	40 (6.4)
Any recurrence	32 (5.1)
Contralateral breast cancer	8 (1.3)
Death	20 (3.2)
Breast cancer	10 (1.6)
Other cancer	4 (0.6)
Cardiovascular disease	1 (0.2)
Liver disease	1 (0.2)
Other cause	1 (0.2)
Unknown	3 (0.5)

### Risk factors for DFS

By univariate Cox-regression analysis, tumor size, ER status, HER-2 status, tumor location, LVI status, Ki-67 index, grade and chemotherapy were significantly associated with DFS (**Table 2**, all p<0.1). Subsequently, multivariate analysis indicated that tumor size (*p*=0.006), tumor location (*p*=0.033), LVI status (*p*=0.028) and Ki-67 index (*p*=0.051) were four independent risk factors for DFS among pN0 BC patients treated with BCS+WBI alone (**Table 3**).

**Table 3:** Cox-univariate and multivariate analysis for DFS.

Characteristics	Univariate analysis		Multivariate analysis	
	HR, 95%	*P* value	HR, 95%	*P* value
Age			–	
**≤50**	1		–	–
**>50**	0.65 (0.38-1.11)	0.12	–	
Tumor size			–	–
**≤2cm**	1		1	
**>2cm**	2.65 (1.52-4.63)	0.0006	2.24 (1.26-4.00)	0.006
ER status,				
** negative**	1		1	
** positive**	0.35 (0.20-0.60)	0.0001	0.74 (0.39-1.05)	0.36
HER-2 status				
** negative**	1		1	
** positive**	2.02 (1.01-4.02)	0.046	1.55 (0.76-3.17)	0.23
Tumor location				
** External**	1		1	
** Centrally or medially**	1.70 (0.99-2.91)	0.055	1.82 (1.05-3.15)	0.033
LVI status				
** negative**	1		1	
** positive**	3.59 (1.88-6.85)	0.0001	2.26 (1.09-4.69)	0.028
Ki-67 index				
**≤14%**	1		1	
**>14%**	3.81 (1.86-7.81)	0.0003	2.37 (1.00-5.62)	0.051
Grade				
** Grade I-II**	1		1	
** Grade III**	3.53 (1.99-6.25)	<0.0001	1.88 (0.94-3.74)	0.073
Histology				
** Others**	1		–	
** IDC**	1.45 (0.58-3.64)	0.43	–	–
Hormonal therapy				
** no**	1		–	
** yes**	0.82 (0.40-1.67)	0.59	–	–
Chemotherapy				
** yes**	1		1	
** no**	2.99 (1.45-6.17)	0.003	0.79 (0.36-1.73)	0.55

### Risk stratification system

**Figure 2:**
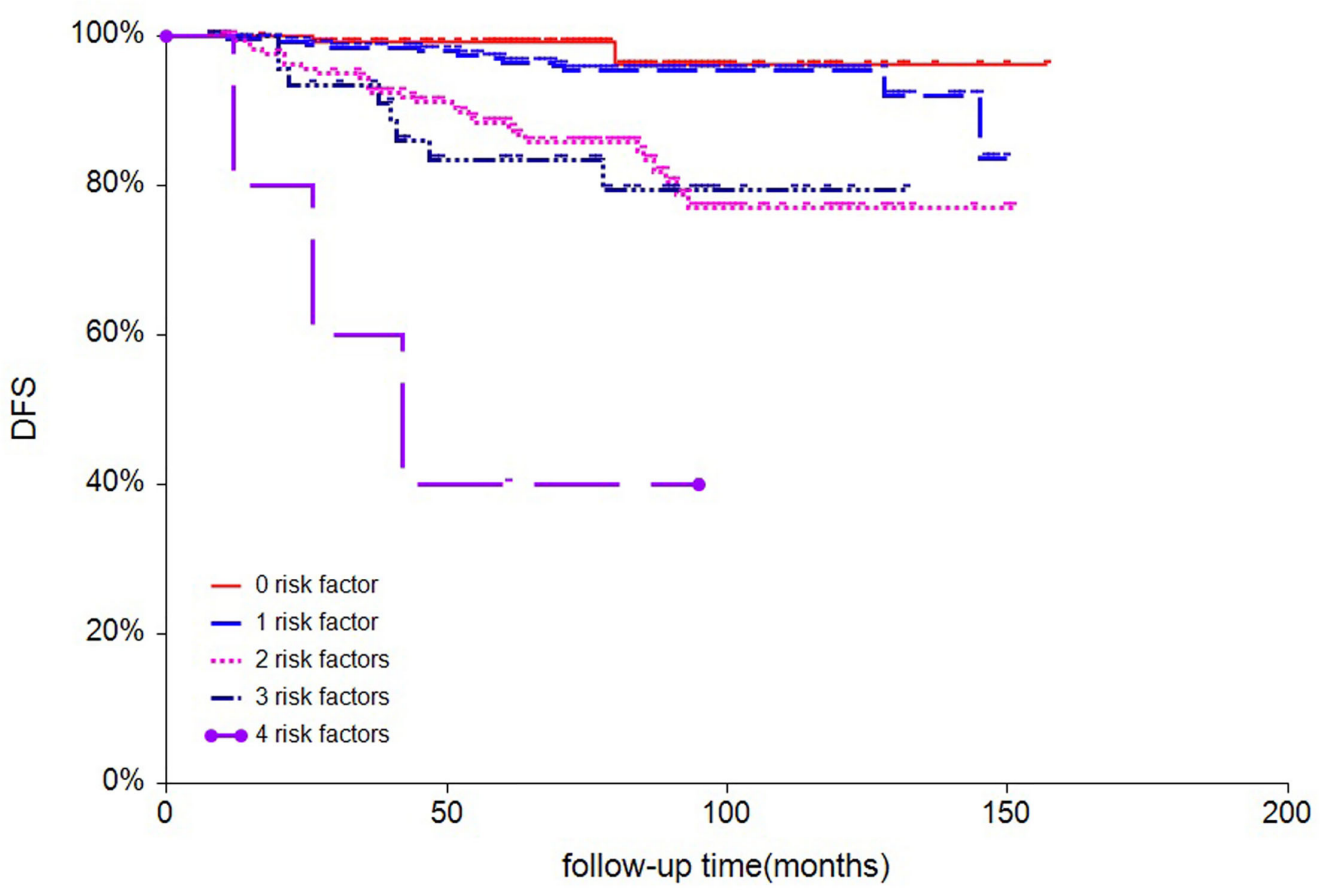
survival analysis of disease-free survival of all 622 patients stratified by numbers of risk factors.

**Figure 3:**
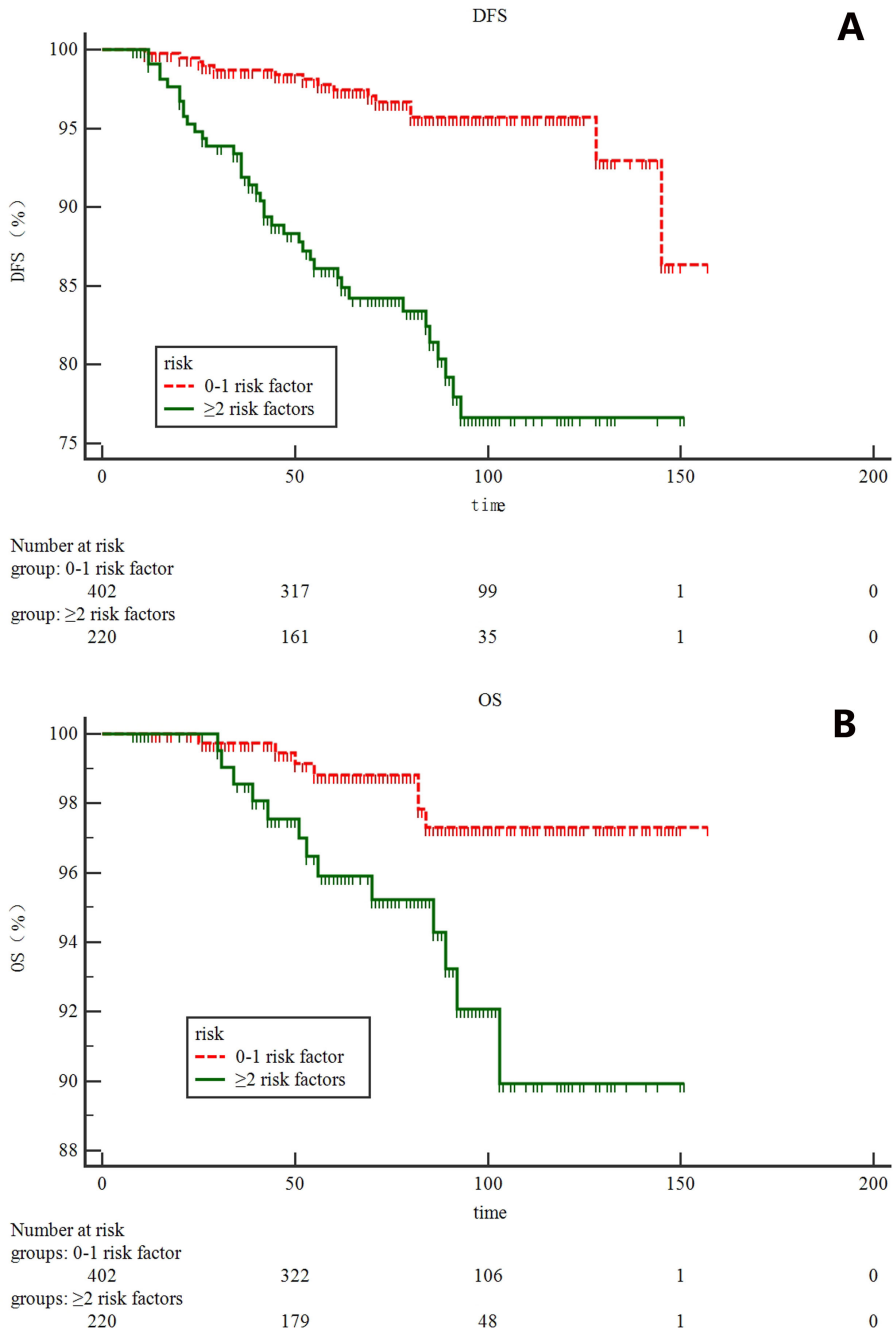
survival analysis for all 622 patients according to risk stratification system: **(A)** disease-free survival; **(B)** overall survival.

According to tumor size, tumor location, LVI status and Ki-67 index, patients were stratified into five subgroups, and 7-year DFS were 96, 95, 85, 79 and 40.0% for patients with 0, 1, 2, 3, and 4 risk factors, respectively (**Figure 2**). Based on the magnitude and differences of DFS between the five subgroups, patients were further stratified into two groups: 402 patients (64.6%) with 0-1 risk factors, and 220 patients (35.4%) with ≥2 risk factors. For patients with 0-1 and ≥2 risk factors, the 7-year DFS rates were 96% and 82.0%, respectively (*p*<0.001) (**Figure 3**). In addition, the 7-year OS for pN0 BC with 0-1 risk factor was significantly higher than those with ≥2 risk factors (97% vs. 95%, p=0.0063, **Figure 3**).

### External validation

**Figure 4:**
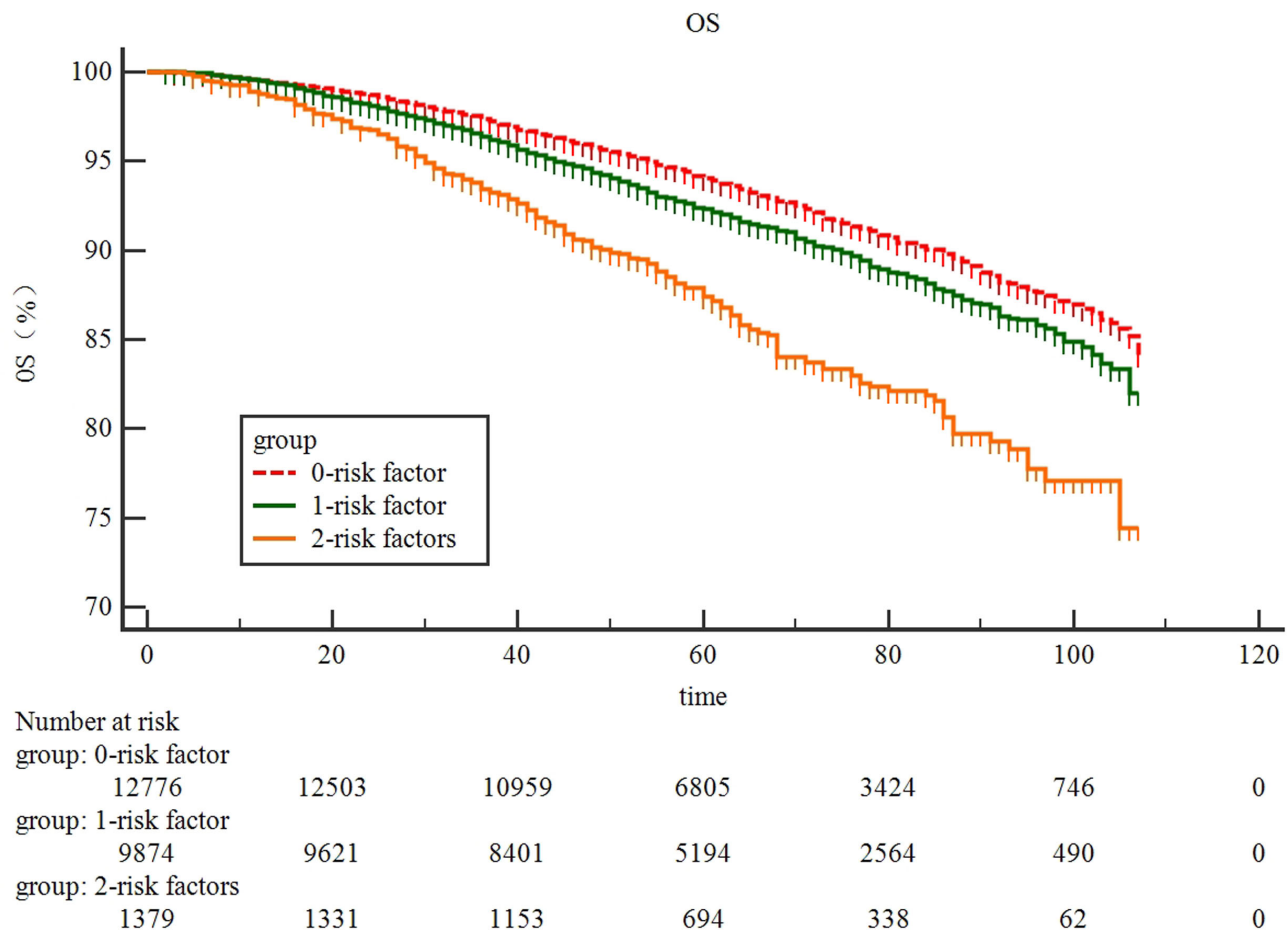
external validation of risk stratification system in 24,029 pN0 patients from SEER database.

By using SEER database, two risk factors including tumor size and tumor location could be obtained. Our result showed that the 7-year OS was 90% and 88% for BC patients with 0-1 risk factor, which was significantly better than those defined as high-risk BC patients (82%, *p*<0.0001, **Figure 4**).

## Discussion

As the EORTC-22922 and MA-20 trials demonstrated that comprehensive RNI including infraclavicular region, supraclavicular area and internal mammary nodes could significantly improved DFS and breast cancer specific survival of early-stage BC (10, 11), we saw a paradigm shift toward comprehensive RNI among BC patients with one to three positive nodes in our single institute (from 16% in 2015 to 83% in 2019, unpublished data). And these two trials led to strongly recommends radiotherapy to comprehensive nodal area for pN1 BC patients according to NCCN guideline. On the other hand, although both clinically high-risk pN0 and pN1 BC were enrolled in the EORTC-22922 and MA-20 trials and sub-group analysis indicated a more decreased survival benefit of DFS (HR 0.55 for N0 vs 0.85 for N1) and OS (HR 0.79 for N0 vs. 0.89 for N1) could be obtained from comprehensive RNI among clinically high risk pN0 BC patients when compared to pN1 group, only 5% of pN0 BC patients after BCS treated with RNI in our institute from 2009-2016. In addition, NCCN guideline only suggest that RNI could be considered in highly selected cases defined by a combination of unfavorable features, but this recommendation was “conditional” and “not strongly” due to heterogenous risk factors for pN0 BC patients and “moderate” quality of evidence. As a result, there is an urgent need to establish a risk stratification system for identifying sub-group of pN0 BC patients who could benefit from comprehensive RNI.

To our best knowledge, the present study was the first study focusing on risk factors for pN0 BC patients after BCS+WBI alone. A total of 622 BC patients were included for analysis. With a median follow-up of 82 months, the 7-year OS and DFS for the entire cohort was 97% and 91%, respectively. By using Cox-multivariate analysis, we finally identified four independent prognostic factors which were associated with an increased risk of DFS after adjusting for ER status, HER-2 status, grade and adjuvant chemotherapy. External validation based on tumor size and tumor location indicated that OS of high-risk pN0 BC was significantly poorer than those presented with 0-1 risk factor.

Prior to the present study, multiple studies had demonstrated that the site of primary tumor may be an important characteristic affecting the prognosis of patients with breast cancer (13–15). Colleoni M. et al (16) found that the risk of relapse for patients with medial presentation was largest for the node-negative cohort and for patients with tumors larger than 2 cm. In consistent with previous results, our result found that tumor location was an independent risk factor for DFS. In addition, tumor size and LVI status were two independent risk factors for DFS among this patient cohorts, which were also used in MA-20 trial to identify clinically high-risk N0 BC patients. Ki-67, a nuclear protein firstly identified by Gerde et al. (17), was associated with cellular proliferation. Inwald E.C. et al (18) performed a large population-based cohort and demonstrated that patients with tumors that had a high-Ki-67-labeling index had both worse DFS and OS than patients with tumors that had low-Ki-67-labeling index, and Ki-67 values >15% seem to have a linear relationship with OS and DFS. In consistent with previous findings, we found that Ki-67 index was another independent risk factor for developing DFS among pN0 BC patients. Based on these four independent risk factors, a scoring system was developed. The 7-year DFS and OS were 97% and 96% for patients with 0-1 risk factors, 95% and 82% for patients with ≥2 risk factors (*p*<0.0001 for DFS, and *p*=0.0063 for OS). By using this risk scoring system, pN0 BC patients after BCS and WBI could be stratifying into two different risk groups and our research team is planning a multi-center randomized trial to further confirm the efficacy of RNI in clinically high risk pN0 BC patients when compared to WBI alone.

The present study had several limitations. First of all, this was a retrospective study, the inherent limitations, such as selection bias, could not be avoided. Secondly, the median follow-up of present study was 82 months, the number of deaths was too small to analyze the multivariable Cox proportional hazards regression analysis for overall survival. Therefore, long-term follow-up for this patient population was still needed. Finally, only tumor size and tumor location could be obtained from SEER database, and the detailed radiation strategy for BC patients was unknown, therefore, more external validations of our risk stratification system were urgently needed.

## Conclusion

The results of this analysis demonstrate that tumor size, tumor location, lymphovascular invasion status and Ki-67 index are four independent risk factors for DFS among pN0 BC patients undergoing BCS and WBI alone. Based on these four risk factors, pN0 BC patients could be stratifying into two different risk groups of DFS and OS. The results of our study support tailored RT decision-making according to individual risks, which needed to be confirmed in further prospective studies.

## Data Availability

The raw data supporting the conclusions of this article will be made available by the authors, without undue reservation.
